# EZH2 facilitates BMI1-dependent hepatocarcinogenesis through epigenetically silencing microRNA-200c

**DOI:** 10.1038/s41389-020-00284-w

**Published:** 2020-11-09

**Authors:** Leibo Xu, Junlong Lin, Wanyu Deng, Weixin Luo, Yipei Huang, Chao-Qun Liu, Fa-Peng Zhang, Yu-Fei Qin, Ping-Pui Wong, Chao Liu

**Affiliations:** 1 Department of Biliary-Pancreatic Surgery, Sun Yat-sen Memorial Hospital, Sun Yat-sen University, Guangzhou, 510120 China; 2 Guangdong Provincial Key Laboratory of Malignant Tumor Epigenetics and Gene Regulation, Sun Yat-sen Memorial Hospital, Sun Yat-sen University, Guangzhou, 510120 China; 3 College of Life Science, Shangrao Normal University, Shangrao, Jiangxi Province China

**Keywords:** Oncogenes, Liver cancer

## Abstract

EZH2, a histone methyltransferase, has been shown to involve in cancer development and progression via epigenetic regulation of tumor suppressor microRNAs, whereas BMI1, a driver of hepatocellular carcinoma (HCC), is a downstream target of these microRNAs. However, it remains unclear whether EZH2 can epigenetically regulate microRNA expression to modulate BMI1-dependent hepatocarcinogenesis. Here, we established that high EZH2 expression correlated with enhanced tumor size, elevated metastasis, increased relapse, and poor prognosis in HCC patients. Further clinical studies revealed that EZH2 overexpression was positively correlated to its gene copy number gain/amplification in HCC. Mechanistically, EZH2 epigenetically suppressed miR-200c expression both in vitro and in vivo, and more importantly, miR-200c post-transcriptionally regulated BMI1 expression by binding to the 3′-UTR region of its mRNA. Furthermore, miR-200c overexpression inhibits the growth of HCC cells in vivo. Silencing miR-200c rescued the tumorigenicity of EZH2-depleted HCC cells, whereas knocking down BMI1 reduced the promoting effect of miR-200c depletion on HCC cell migration. Finally, combination treatment of EZH2 and BMI1 inhibitors further inhibited the viability of HCC cells compared with the cells treated with EZH2 or BMI1 inhibitor alone. Our findings demonstrated that alteration of EZH2 gene copy number status induced BMI1-mediated hepatocarcinogenesis via epigenetically silencing miR-200c, providing novel therapeutic targets for HCC treatment.

## Introduction

Hepatocellular carcinoma (HCC) is the sixth most common malignancy and the third leading cause of cancer mortality worldwide^1^. The HCC patient survival rate remains poor mainly owing to the poor diagnosis and limited treatment options. Surgical resection or liver transplantation are the mainstay of the HCC therapeutic strategies. Although sorafenib, a multiple kinase inhibitor, has been currently used for treating unresectable HCC patients, its usage as an adjuvant therapy remains controversial^2^. Therefore, there is an urgent need to explore the molecular pathogenesis of HCC in order to discover improved diagnosis methods and therapeutic strategies.

Recent studies have shown that polycomb repressive complexes (PRC) play an important role in maintaining stem cell behavior and cancer biology^3,4^. They consist of two major classes, PRC1 and PRC2, that can collaboratively regulate gene expression by modifying chromatin structures^5^. For instance, EZH2, an enzymatic component of PRC2, can silence its target genes by inducing the trimethylation of lysine 27 on histone 3 (H3K27me3)^6,7^. Afterwards, PRC1, composed of BMI1, RING1 and RING2, recognizes the H3K27me3 mark produced by the PRC2 complex and then mono-ubiquitinates K119 on histone 2 A to stabilize/maintain long-term gene silencing^8,9^. Owing to the important roles of EZH2 and BMI1 in epigenetic regulation, they are often overexpressed in cancer cells and are required for self-renewal of stem cells^10,11^. Indeed, accumulating evidences suggest that increased activities of PCR1 and PCR2 are oncogenic as measured by xenograft tumor growth, cell invasion, maintenance of cancer stemness, and metastasis^12–14^. At present, very little known about the molecular mechanisms that regulate EZH2 and BMI1 expression in HCC.

MicroRNAs are a class of 22-nucleotide single-stranded noncoding RNA that play critical role in a variety of diseases including carcinogenesis^15,16^. They can regulate gene expression by binding to 3′-UTR of their target messenger RNA^17^. It has been proposed that miRNAs coordinate the regulation of PRC1 and PRC2 activities in prostate cancer and neural stem/progenitor cells^9,18^. However, the underlying mechanism of their functional coordination has not been fully elucidated in HCC. In this study, we sought to explore the regulatory axis between EZH2 and BMI1, and determine whether microRNAs would regulate the synergy between them in order to drive hepatocarcinogenesis.

## Results

### High EZH2 expression strongly correlates with poor prognosis in HCC patients

**Fig. 1:**
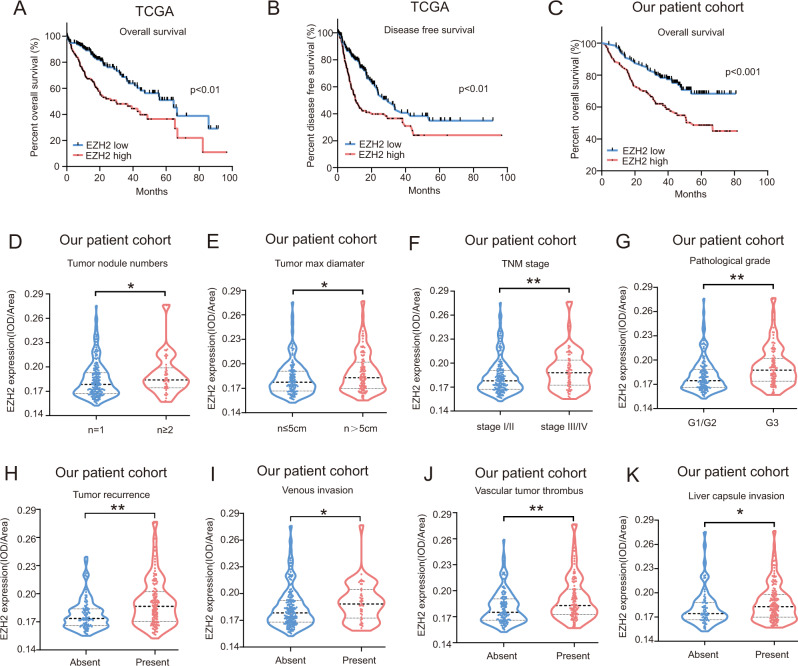
High expression of EZH2 strongly correlates with poor prognosis in HCC patients. Analysis of EZH2 expression in human hepatocellular carcinomas from TCGA data set (*n* = 366 HCC patients) and our patient cohort (*n* = 240 HCC patients). **a**, **b** The HCC patients with high EZH2 expression strongly correlated with poor postoperative overall survival (*n* = 365 HCC patients, TCGA data set) and disease-free survival time (*n* = 313 HCC patients, TCGA data set). **c** Immunohistochemistry (IHC) study of EZH2 protein expression in human HCC tissues from our patient cohort validated that the HCC patients with high EZH2 expression strongly correlated with poor postoperative overall survival (*n* = 240 HCC patients, our patient cohort). **d**–**k** IHC study validated that high expression of EZH2 stratified the HCC patients into those with increased tumor nodule numbers **d**, enhanced tumor size **e**, elevated TNM stage **f**, advanced pathological grade **g** as well as increased tumor recurrence **h**, enhanced venous invasion **i**, increased vascular tumor thrombus **j** and elevated liver capsule invasion **k** in HCC patients (*n* = 240 HCC patients, our patient cohort). Each sample on the violin plots represents individual patient data. *p < 0.05, **p < 0.01. **a**–**c** Log-rank (Mantel–Cox) test. **d**–**k** Mann–Whitney test.

Previous studies suggest that EZH2 has an important role in cancer development and progression^19,20^. To determine its oncogenic role in HCC, we first examined the expression level of EZH2 in multiple cancers by analyzing GEPIA (Gene Expression Profiling Interactive Analysis) data set and RNA-seq data set retrieved from The Cancer Genome Atlas (TCGA) database. Intriguingly, we observed that the expression of EZH2 was upregulated in different cancers (Figure S1), indicating the dysregulation of EZH2-mediated signaling pathway was a common phenomenon in tumorigenesis. Importantly, high EZH2 expression was strongly associated with poor overall survival (OS) (Fig. 1a) and worse disease-free survival (DFS) (Fig. 1b) in HCC patients. Further clinical studies revealed that high expression of EZH2 stratified the HCC patients into those with elevated neoplasm disease stage (Figure S2A), enhanced cancer progression (Figure S2, B), and high histologic grade (Figure S2C). To validate these observations, we performed immunohistochemistry (IHC) analysis of EZH2 expression in our cohort of HCC patients, indicating that high expression of EZH2 was strongly associated with poor OS (Fig. 1c) and increased tumor nodule numbers (Fig. 1d), enhanced tumor size (Fig. 1e), elevated cancer progression (Fig. 1f), high pathological grade (Fig. 1g), increased tumor recurrence (Fig. 1h) as well as increased venous invasion (Fig. 1i), enhanced vascular tumor thrombus (Fig. 1J), and elevated liver capsule invasion (Fig. 1k). Overall, our data indicated that EZH2 may potentially determine HCC development and progression, which prompted us to further explore its oncogenic role.

### Alteration of EZH2 gene copy number status strongly associates with its mRNA expression level in HCC patients

**Fig. 2:**
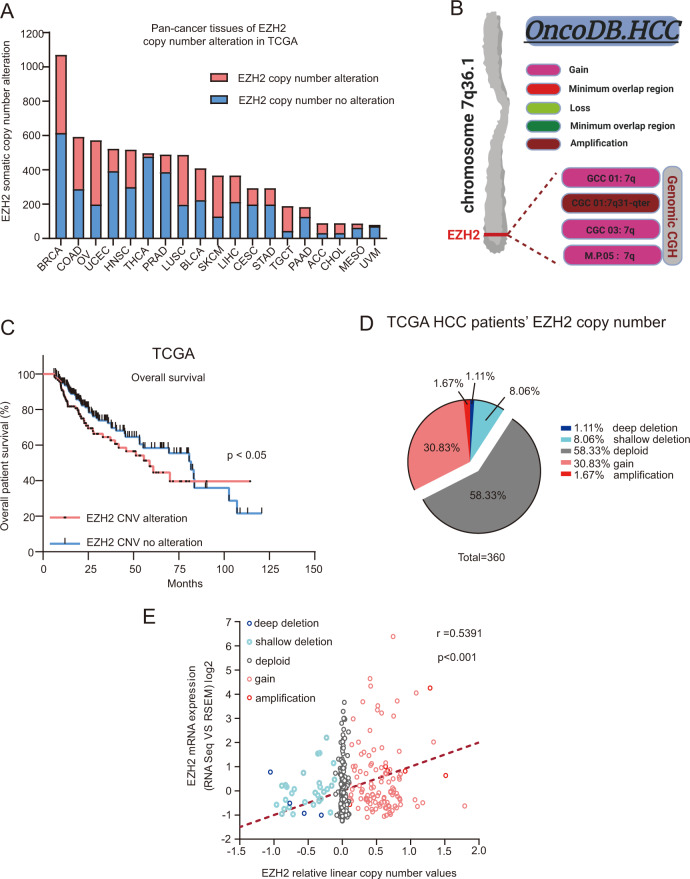
EZH2 gene copy number variation affects its expression in HCC patients. **a** Analysis of TCGA data set revealed a common phenomenon of the somatic EZH2 gene copy number alteration across a panel of cancer types. **b** Analysis of oncogenomic database indicated a significant EZH2 gene copy number gain/amplification (the region of chromosome 7q36.1) in HCC patients. **c** The alteration of EZH2 gene copy number status was associated with poor postoperative overall survival in HCC patients. **d** Pie chart indicated the status of EZH2 copy number variation in HCC patients (*n* = 360 HCC patients, TCGA data set). **e** Pearson correlation study showed that the mRNA expression of EZH2 was positively correlated with its gene copy number values (*n* = 360 HCC patients, TCGA data set). The gene copy number, levels of mRNA and clinical data were obtained through the cBioportal analysis platform. Bar chart represents mean±S.E.M. *p < 0.05, **p < 0.01. **c** Gehan–Breslow–Wilcoxon test. **e** Pearson correlation.

To explore the molecular mechanism of EZH2 overexpression in HCC, we analyzed the copy number variation of EZH2 gene in a PAN-cancer panel using the TCGA data set. Interestingly, alteration of EZH2 gene copy number was observed in multiple cancer types (Fig. 2a). In addition, we performed comparative genome hybridization (CGH) studies using the HCC oncogenomic database, indicating that increased chromosome 7q36.1, where EZH2 located, copy number gain/amplification was observed in HCC patients (Fig. 2b). Indeed, analysis of the TCGA data set revealed that the HCC patients with EZH2 gene copy number variation was associated with poor postoperative OS (Fig. 2c). Further bioinformatics analysis of the TCGA data set revealed that >32% human HCC patients had EZH2 gene copy number gain/amplification, whereas the expression level of EZH2 was positively correlated with its copy number values (Fig. 2d, e). Therefore, our result indicated that the alteration of EZH2 gene copy number directly correlated to its mRNA expression level in HCC patients.

### Knockdown of EZH2 suppresses tumorigenesis in HCC cell lines

**Fig. 3:**
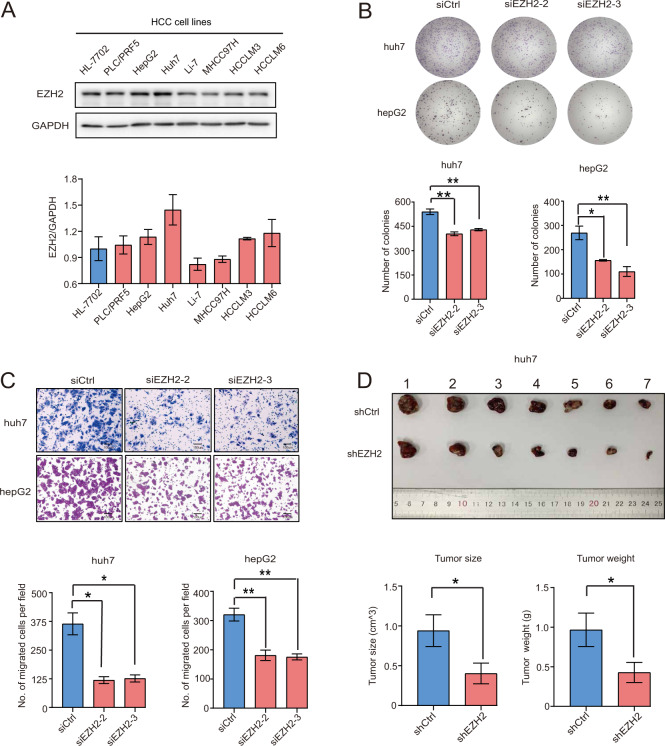
Depletion of EZH2 impaired the proliferation, migration, and tumorigenic ability in HCC cells. **a** The protein level of EZH2 in an immortalized liver cell line (HL-7702) and seven HCC cell lines were measured by western blotting. GAPDH was used as a loading control. Relative densitometry was calculated by Image J analysis software. **b** Colony formation assay of the huh7 and hepG2 cell transiently transfected with siCtrl, siEZH2-2, siEZH2-3. **c** Transwell migration assay of the huh7 and hepG2 cells transiently transfected with siCtrl, siEZH2-2, siEZH2-3. **d** Representative image of gross tumors harvested from the nude mice subcutaneously injected with huh7 cells stably transfected with shCtrl or shEZH2 is given. Bar charts show the tumor weight and size of each group. Bar charts represent mean±S.E.M. *p < 0.05, **p < 0.01. **b**–**c** One-way ANOVA. **d** Student’s *t* test. Scale bars in **c** 100 μm.

We performed western blotting analysis of the EZH2 expression in an immortalized human liver cell HL-7702 and a panel of HCC cell lines, indicating that the expression level of EZH2 was higher in huh7 and hepG2 cell lines compared to the rest of HCC cell lines and HL-7702 (Fig. 3a). Therefore, we selected huh7 and hepG2 cell lines to establish the EZH2 knockdown models for our studies. huh7 and hepG2 cells were transiently transfected with two independent siRNA sequences. The knockdown efficiency of EZH2 in these two cell lines were confirmed by western blotting, indicating that both siRNA sequences achieved >50% of silencing efficiency (Figure S3A and B). Meanwhile, depletion of EZH2 in HCC cells significantly decreased the expression level of H3K27me3 as compared with the scramble control, indicating that EZH2 probably regulated epigenome via transcriptional repressive H3K27me3 in HCC. (Figure S3C and D). As our clinicopathological association study indicated that high EZH2 expression strongly correlated with proliferative and aggressive clinicopathological features in HCC patients, we therefore performed colony formation, transwell migration, and cell proliferation assays with the EZH2-depleted cells and scramble control cells. As expected, knockdown of EZH2 markedly impaired the colony formation and migration ability of HCC cells as compared with the scramble control (Fig. 3b, c). Furthermore, CKK-8 cell proliferation assay showed that silencing EZH2 inhibited the proliferation of HCC cells compared with the scramble control (Figure S3E). To explore whether EZH2 determined the tumorigenicity of HCC cells in vivo, nude mice were subcutaneously injected with huh7 cells stably transfected with either scramble control vector (Lv-Ctrl) or shRNA targeting EZH2 (Lv-shEZH2). Our result indicated that depletion of EZH2 significantly reduced the size and weight of tumors as compared with the control group (Fig. 3d). These results indicated that EZH2 plays an important role in hepatocarcinogenesis via an unknown mechanism.

### Silencing miR-200c rescues the sphere formation and migration deficits in EZH2-depleted HCC cells

**Fig. 4:**
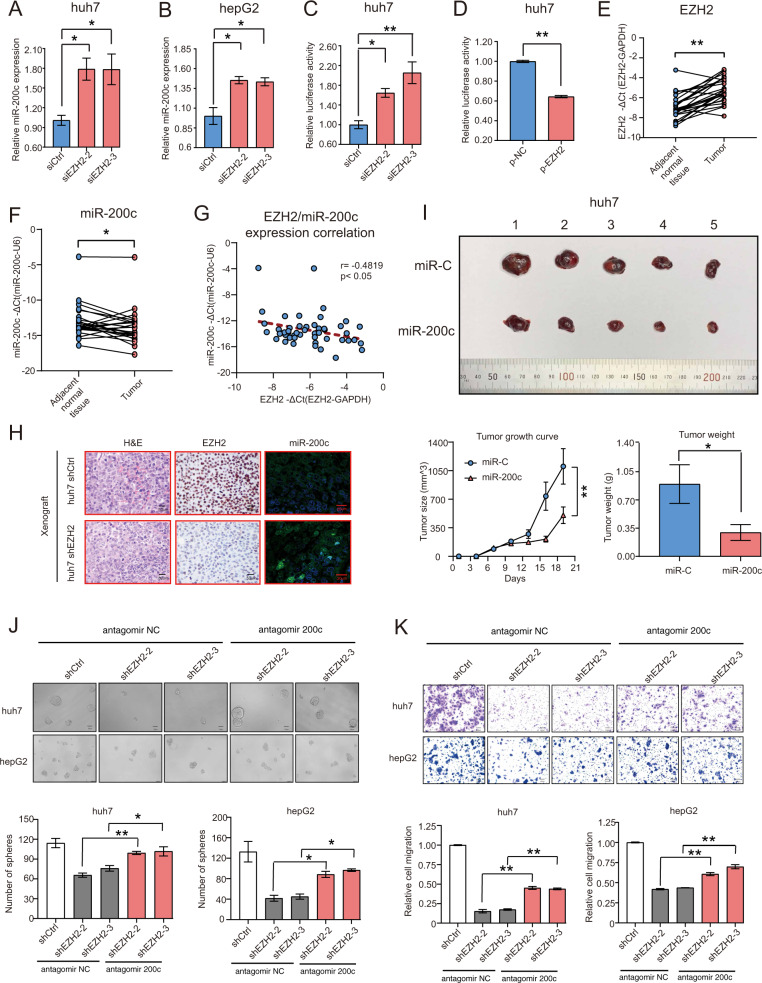
Knockdown of miR-200c rescues the proliferation, tumorigenicity, and migration deficits in EZH2-depleted HCC cells. **a**, **b** RT-qPCR analysis of the miR-200c expression in huh7 and hepG2 cells transfected with either siCtrl, siEZH2-2, or siEZH2-3. **c** Luciferase reporter activity of the miR-200c promoter in huh7 cells transfected with either siCtrl, siEZH2-2 or siEZH2-3. **d** Luciferase reporter activity of the miR-200c promoter in huh7 cells transfected with control vector (p-NC) or EZH2 overexpression vector (p-EZH2). **e** RT-qPCR analysis of the mRNA expression level of EZH2 in paired HCC tumor tissues and adjacent normal tissues (*n* = 25 HCC patients). **f** RT-qPCR analysis of the miR-200c expression in paired HCC tumor tissues and paired adjacent normal tissues (*n* = 25 HCC patients). U6 was used as internal reference gene. **g** Pearson correlation analysis of the EZH2 and miR-200c expression in paired HCC tumor tissues and adjacent normal tissues (*n* = 25 HCC patients). **h** Representative images of H&E-stained sections, EZH2 immunohistochemically stained sections, miR-200c fluorescence in situ hybridization (FISH) derived from the subcutaneous tumors arising from shCtrl or shEZH2 stably transfected huh7 cells are given. **i** Representative image of gross tumors harvested from the nude mice subcutaneously injected with huh7 cells stably transfected with either vector control (miR-C) or miR-200c lentivirus (miR-200c) is given. Overexpression of miR-200c in huh7 cells significantly inhibited the tumor growth and weight as compared with vector control group. **j** Representative images of the sphere formation assays derived from shCtrl, shEZH2-2, or shEZH2-3 stably transfected huh7 and hepG2 cells after transiently transfected with antagomir-NC or antagomir-200c are given. Bar charts represent the number of spheres per group in huh7 and hepG2 cells respectively. **k** Representative images of the relative migration of shCtrl, shEZH2-2, or shEZH2-3 stably transfected huh7 and hepG2 cells after transiently transfected with antagomir-NC or antagomir-200c are given. Bar charts represent the relative cell migration for each group in huh7 and hepG2 cells, respectively. The data were obtained from three to four independent experiments and presented with mean±S.E.M. *p < 0.05, **p < 0.01. **a**, **c**, **j**–**k** One-way ANOVA. **b** Kruskal–Wallis test. **d** Unpaired *t* test. **e**, f paired *t* test. **g** Pearson correlation. **i** Two-way ANOVA and Student’s *t* test. Scale bars in **j** were 50 μm (huh7) and 100 μm (hepG2). Scale bars in **k** 100 μm.

Emerging evidences have demonstrated that miRNAs play a prominent role in the occurrence and progression of malignant tumors, whereas EZH2 can epigenetically regulate miRNAs in many malignant tumors^21–25^. Indeed, recent studies reported that EZH2 can negatively regulate miR-200c expression in renal cancer and prostate carcinoma via the epigenetic regulation^9,26^. To validate whether this is the case in HCC, we transfected HCC cells with either scramble or EZH2 targeting siRNA, indicating that depletion of EZH2 dramatically enhanced miR-200c expression in both huh7 and hepG2 cells (Fig. 4a, b). In the meantime, EZH2 was involved in the regulation of miR-200c promoter activity, as knockdown of EZH2 generated a higher luciferase activity, whereas overexpression of EZH2 generated a lower luciferase activity of miR-200c promoter compared with the scramble control in huh7 (Fig. 4c, d). To prove the clinical significance of our finding, we next examined the endogenous expression of EZH2 and miR-200c by RT-qPCR in 25 paired frozen HCC tissues and adjacent normal liver tissues. The results showed that the HCC tissues had high expression level of EZH2 and low expression level of miR-200c when compared with the adjacent normal liver tissues (Fig. 4e, f). Further statistical analysis indicated that there was a negative correlation between EZH2 and miR-200c in HCC tissues (Fig. 4g). Consistent with our finding, both IHC and fluorescence in situ hybridization (FISH) analysis of the subcutaneous tumors also showed high EZH2 and low miR-200c expression in the control group tumors, whereas EZH2 depletion significantly enhanced the expression of miR-200c in tumors as compared with the control group (Fig. 4h).

As EZH2 determines HCC progression and metastasis, we next sought to investigate its role in miR-200c mediated tumorigenesis. It has been reported that miR-200c was a tumor suppressor microRNA in many cancers, such as colorectal cancer, renal cell carcinoma^27,28^. However, the in vivo role of miR-200c in HCC tumor cell growth has yet to be elucidated. We therefore subcutaneously injected huh7 cells stably transfected with either vector control or miR-200c lentivirus into nude mice. Our results showed that overexpression of miR-200c in huh7 cells significantly inhibited tumor growth and developed smaller tumors as compared with scramble group (Fig. 4i). Based on this in vivo data, we hypothesized that EZH2 exerted its tumor-promoting function in HCC via down regulating miR-200c expression. To test this hypothesis, we performed sphere formation and transwell migration assays with the scramble or EZH2 targeting shRNA stably transfected huh7 and hepG2 cells that were transiently transfected with antagomir-200c (an antisense inhibitor of miR-200c) or non-silencing control (antagomir-NC). Our results indicated that knockdown of EZH2 in HCC cells significantly inhibited the tumor sphere formation and migration, whereas antagomir-200c transfection partly rescued the effect caused by EZH2 knockdown, suggesting that EZH2 promoted liver cancer progression at least in part by restraining the expression of miR-200c (Fig. 4j, k).

### EZH2 overexpression enhances BMI1-dependent hepatocarcinogenesis via epigenetically silencing microRNA-200c

**Fig. 5:**
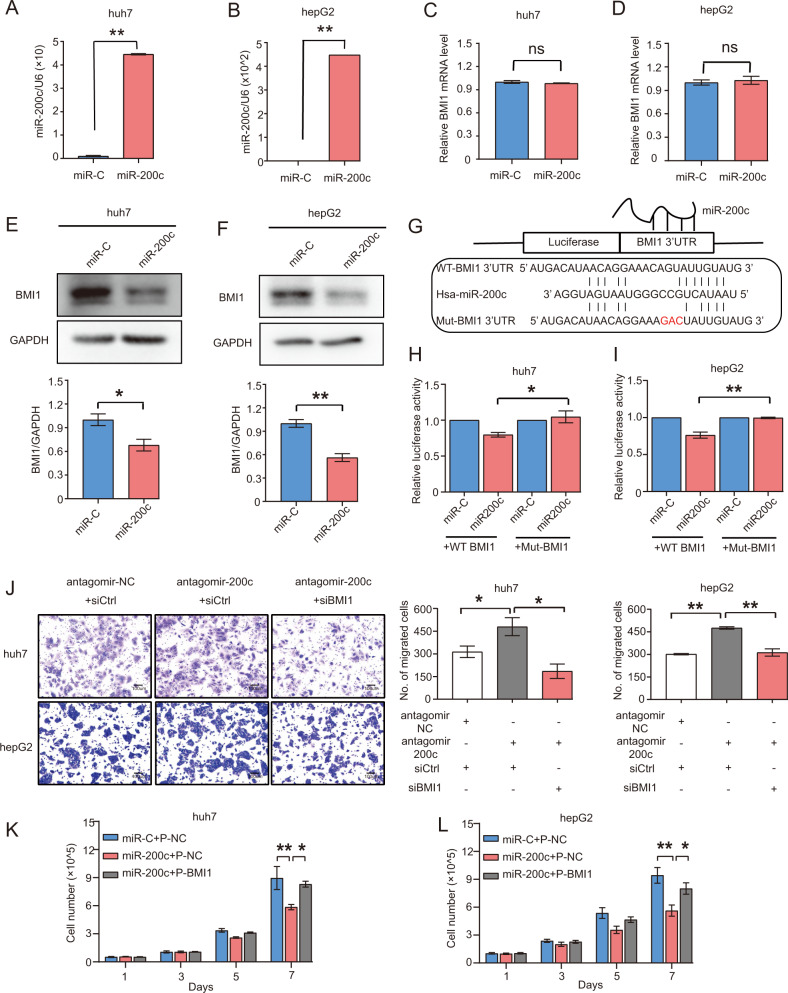
EZH2 regulates BMI1-dependent hepatocarcinogenesis via epigenetic repression of miR-200c. **a**, **b** RT-qPCR analysis of the miR-200c expression in huh7 and hepG2 cells after transiently transfected with scramble control (miR-C) or miR-200c mimics. **c**, **d** RT-qPCR analysis of the BMI1 expression in huh7 and hepG2 cells after transiently transfected with scramble control (miR-C) or miR-200c mimics. **e**, **f** Western blotting of the protein level of BMI1 in huh7 and hepG2 cells after transiently transfected with scramble control (miR-C) or miR-200c mimics. GAPDH was used as a loading control. All the western blots were performed at three independent replicates. Relative densitometry was calculated by Image J analysis software. **g** Representation of seeding region matching between miR-200c and BMI1 3’UTR. **h**–**i** Luciferase reporter assay of the huh7 **h** and hepG2 **i** cells after co-transfected with the psicheck2 dual luciferase reporter vector containing the insert of either the predicted wild-type-3’-UTR (WT-BMI1) or mutated-3’-UTR region (Mut-BMI1) of BMI1 together with scramble control (miR-C) or miR-200c mimics. **j** Silencing miR-200c by antagomir-200c increased the migration of huh7 and hepG2 cells, which could be rescued by silencing BMI1 expression. **k**–**l** Cell counting experiment of the huh7 **k** and hepG2 cells **l** after stably transfected with either vector control or miR-200c lentivirus together with transient transection of control vector (P-NC) or BMI1 overexpression vector (P-BMI1). Overexpression of miR-200c inhibited the proliferation of huh7 and hepG2 cells, whereas BMI1 overexpression reversed the proliferation deficit caused by miR-200c overexpression in both huh7 and hepG2 cells. The data were obtained from three independent experiments and presented with mean ± S.E.M. *p < 0.05, **p < 0.01. ns, no significant difference. **a**–**f**, **h**–**i** Student’s *t* test. **j** One-way ANOVA. **k**–**l** Two-way ANOVA. Scale bars in **j** 100 μm.

It is well known that microRNAs control cellular functions by regulating specific downstream genes^29^. We explored the putative molecular targets of miR-200c by using the TargetScan software. Among the potential targets, BMI1 was one of the top targets because of the 3′-untranslated region (3′-UTR) of BMI1 containing a conserved miR-200c seed-matching sequence. In addition, our previous work indicated that silencing BMI1 impairs the proliferation and invasion of HCC cells^30^. We therefore hypothesized that miR-200c exerted its tumor suppressive function by reducing BMI1 expression in HCC. To test our hypothesis, huh7 and hepG2 cell lines were transfected with either miR-200c mimics, scramble control (miR-C), antagomir-200c or antagomir-non-silencing control (antagomir-NC), whereas the overexpression or knockdown of miR-200c was confirmed by RT-qPCR (Fig. 5a, b, Figure S4A and B). The protein and mRNA level of BMI1 in both huh7 and hepG2 transfected with miR-200c mimics were checked by western blot and RT-qPCR. Interestingly, the protein level of BMI1 was significantly reduced in the miR-200c mimics transfected cells as compared to the scramble control, but there was no difference between them at the mRNA level of BMI1 (Fig. 5c–f). Also, depletion of miR-200c by antagomir-200c increased the protein level of BMI1, whereas it had no effect on BMI1 expression at mRNA level in HCC cells (Figure S4C-F). This result indicated that miR-200c repressed BMI1 expression at protein level via post-transcriptional mechanism. To determine whether miR-200c directly interacted with the 3′-UTR binding site of BMI1, we performed dual luciferase reporter assays using the psicheck2 dual luciferase reporter vector containing an insert of either the wild-type (WT-BMI1) or mutant-type (Mut-BMI1) BMI1 3′-UTR binding site of miR-200c (Fig. 5g). Then, huh7 and hepG2 cells were co-transfected with either the WT-BMI1 psicheck2 vector or Mut-BMI1 psicheck2 vector together with either the miR-200c mimics or scramble control (miR-C) for 48 h. Results showed that miR-200c overexpression decreased the luciferase activity in both huh7 and hepG2 cell lines transfected with the vector containing WT-BMI1, but not the one with Mut-BMI1 (Fig. 5h, i). We also included the luciferase reporter assay using miR-200c antagomirs in hepG2. The results showed inhibition of miR-200c increased the luciferase activity in hepG2 after transfected with the vector containing WT-BMI1, but not the vector containing Mut-BMI1(Figure S4G). Taken together, our data indicated that BMI1 was a direct downstream target of miR-200c in HCC cells. As BMI1 was negatively regulated by miR-200c, we then explored whether miR-200c suppressed tumorigenesis via BMI1. Interestingly, depletion of miR-200c by antagomir-200c increased the migration ability of both huh7 and hepG2 cells, whereas the effect could be rescued by silencing BMI1 expression (Fig. 5j). In addition, both CCK8 proliferation assay and cell counting experiments showed that overexpression of miR-200c inhibited the growth of huh7 and hepG2 cells on day 7, while BMI1 overexpression significantly reversed the proliferation deficit caused by miR-200c overexpression in huh7 and hepG2 cells (Fig. 5k, l, Figure S4H-I). Collectively, our data indicated that miR-200c exerted its tumor suppressive function at least in part by restraining BMI1 expression.

### Combination treatment of EZH2 and BMI1 inhibitors significantly prohibits the viability of HCC cells

**Fig. 6:**
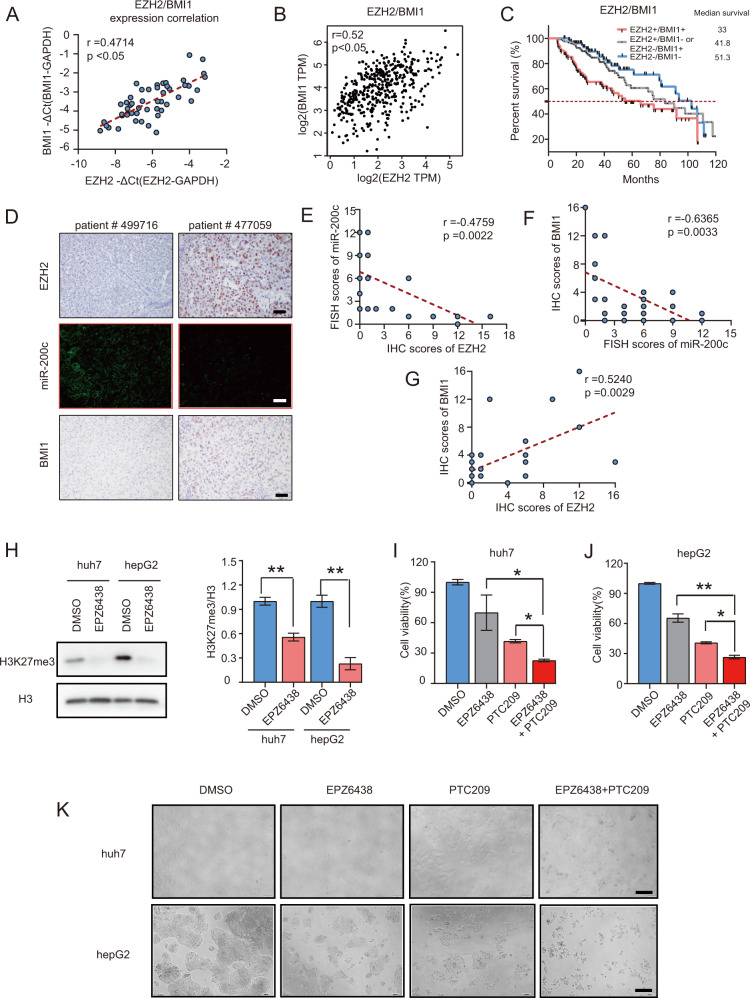
Combined treatment of EZH2 and BMI1 inhibitors prohibits HCC cell proliferation. **a** Pearson correlation analysis of the mRNA expression level of EZH2 and BMII in 25 pairs of HCC tumor tissues and adjacent normal tissues. **b** Pearson correlation analysis of the mRNA expression of EZH2 and BMI1 in HCC tumor tissues in GEPIA data set. **c** High expression of EZH2 and BMI1 strongly associated with poor overall survival in HCC patients (*n* = 283 HCC patients). **d**–**g** Immunohistochemistry and FISH analysis of EZH2, BMI1, and miR-200c expression in tumor sections from 25 HCC patients. Representative images are given. The expression level of EZH2, BMI1, and miR-200c were calculated by multiplying the proportion and intensity. Proportion scores were assigned as follow: <10% = 0, 10–25% = 1, 26–50% = 2, 51–75% = 3 and >75% = 4; intensity scores were assigned as follow: 0 = no staining, 1 = week, 2 = moderate, 3 = strong and 4 = significantly strong. **e**–**g** Correlation studies between the expression of EZH2/BMI1 and miR-200c. **h** Treatment with EZH2 inhibitor EPZ6438 repressed EZH2-mediated H3K27me3 expression in both huh7 and hepG2. Bar chart represents the expression level of H3K27me3 from three independent experiments. **i**–**j** CCK8 assays of the HCC cells treated with either DMSO, EZH2 inhibitor EPZ6438 (32 μM), BMI1 inhibitor PTC209 (400 nM) or EPZ6438 and PTC209 combination in huh7 **i** and hepG2 **j**. **k** Representative bright field pictures of the HCC cells treated with either DMSO, EPZ6438, PTC209, or EPZ6438 and PTC209 combination are given. The data were obtained from three independent experiments and presented with mean ± S.E.M. *p < 0.05, **p < 0.01. **a**–**b**, **e**–**g** Pearson correlation. **c** Log-rank (Mantel–Cox) test. **h** Student’s *t* test. **i**–**j** One-way ANOVA. Scale bars in **d** 50 μm and **k** 100 μm.

**Fig. 7:**
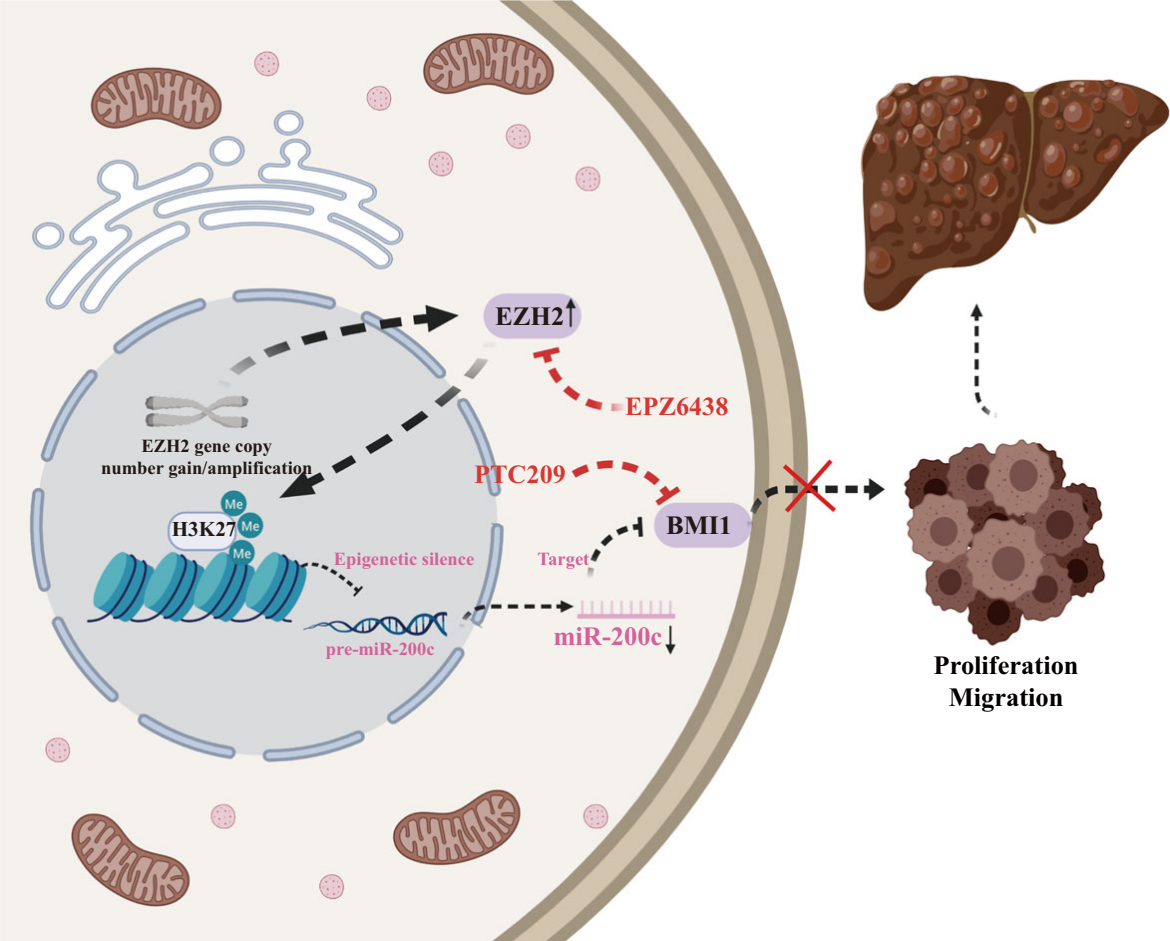
Schematic representation of the mechanistic role of EZH2-mediated epigenetic repression of miR-200c in activating BMI1-dependent tumorigenesis. Alteration of EZH2 gene copy number status up regulates its expression level in HCC, which in turn epigenetically silences miR-200c expression by binding to its promoter sequence. As a result, downregulated miR-200c can enhance BMI1 expression to stimulates tumor cell proliferation and migration in HCC patients. Importantly, treatment with an EZH2 inhibitor (EPZ6438) or BMI1 inhibitor (PTC209) inhibits the viability of HCC cells. Overall, our result demonstrates a new mechanism whereby the EZH2-miR-200c-BMI1 signaling axis determines hepatocarcinogenesis.

Although the regulatory interaction between EZH2 and BMI1 via miRNAs have been demonstrated in prostate cancer^9^, we first demonstrated the importance of EZH2-miR-200c-BMI1 signaling axis in regulating hepatocarcinogenesis. To further validate the importance of this signaling axis, we sought to investigate the correlation between EZH2 and BMI1 expression in HCC patients. RT-qPCR results of HCC tissues showed that the mRNA expression levels of EZH2 and BMI1 were positively correlated (Fig. 6a). Analysis of GEPIA data set also indicated that the expression of EZH2 and BMI1 was positively correlated in HCC patients, whereas high expression of EZH2 and BMI1 stratified the HCC patients into those with the worst OS (Fig. 6b, c). Further IHC and FISH examination of our HCC patient cohort confirmed that the expression of EZH2 or BMI1 were negatively correlated with miR-200c, whereas the expression of EZH2 and BMI1 was positively correlated in our patient cohort. (Fig. 6d–g). To investigate the therapeutic efficacy of targeting EZH2 and BMI1 by small molecular inhibitors, we treated huh7 and hepG2 cells with either an EZH2 inhibitor EPZ6438, a BMI1 inhibitor PTC209 or combination of the above. IC_50_ experiments indicated that the EPZ6438 and PTC209 inhibitor each significantly inhibited the proliferation of huh7 with IC_50_ doses at 57 μM and 435 nM, respectively, and for the hepG2 at 39 μM and 405 nM, respectively. (Figure S5A-D). Western blot confirmed that the EPZ6438 treatment significantly downregulated the expression of H3K27me3 and EZH2 activity in both huh7 and hepG2 cell lines (Fig. 6h). Importantly, both CCK8 assay and morphological studies revealed that combination treatment of EPZ6438 and PTC209, using their suboptimal doses (<IC_50_ doses), significantly inhibited the viability of huh7 and hepG2 cells compared with the cells treated with either DMSO, EPZ6438, or PTC209 alone (Fig. 6i–k). In conclusion, combining EZH2 and BMI1 inhibitors may be a promising therapeutic strategy for HCC treatment (Fig. 7).

## Discussion

Epigenetic regulation, include histone modification, DNA methylation, noncoding RNA, has significant impacts on determining genes expression status and cell fate^31^. Among all the epigenetic regulators, EZH2 is a histone methyltransferase responsible for the epigenetic regulation of genes involved in stem cell biology and developmental processes^8^. However, conflicting results about its oncogenic roles have been reported. Indeed, EZH2 expression has been shown to regulate cancer growth and metastasis in laryngeal squamous cells carcinoma, renal cell carcinoma, melanoma, and pancreatic cancer^7,26,32,33^, whereas other studies show that EZH2 expression improves chemosensitivity in acute myelocytic leukemia^34^ and functions as a tumor suppressor in *kras*-driven lung adenocarcinoma^35^. These conflicting results prompt us to examine its role in hepatocarcinogenesis. We therefore provide clinical evidence from the GEPIA, TCGA data set and our patient cohort, indicating that high EZH2 expression strongly correlates with increased tumor nodules numbers, enhanced tumor size, advanced TNM stage, high pathological grade and increased relapse as well as increased venous invasion, vascular tumor thrombus, and liver capsule invasion. We also provide clinical evidence and animal work indicating the correlation between miR-200c and EZH2/Bmi1 expression. In addition, we demonstrate, for the first time, that overexpression of miR-200c in huh7 cells significantly inhibits the tumor growth and weight as compared with control group, confirming its tumor suppressor role in HCC mouse model. Our result further demonstrates that the EZH2/miR-200c/Bmi1 signaling axis determines the proliferation and migration of HCC cells. Together, these findings suggest that EZH2 overexpression may have an important role in hepatocarcinogenesis.

Although EZH2 has been shown to be frequently upregulated in different cancers^25,36^, the underlying mechanism of this upregulation has not yet been clearly elaborated in HCC. Since chromosome aberration has shown to be strongly correlated with mRNA dysregulation in cancer development^37^, we therefore investigate the oncogenomic database of HCC patients (*OncoDB.HCC*), indicating that chromosome 7q36.1, where EZH2 located, gain/amplification is prevalent in four CGH studies. In addition, recent studies have reported that the EZH2 gain/amplification strongly associates with increased EZH2 mRNA expression in follicular lymphoma^38^ and melanoma^32^. After an integrative analysis of the TCGA data sets, we discover that the EZH2 somatic copy number alteration frequently occurs in different types of cancers. In particular, the HCC patients with EZH2 gene copy number alteration are significantly associated with poor survival. More importantly, the EZH2 gain/amplification is observed in >32% of the total HCC patients, while the expression level of EZH2 is positively correlated with EZH2 gene copy numbers. Therefore, this may explain the reason behind EZH2 overexpression in HCC patients.

Previous studies have indicated that EZH2 can epigenetically silence microRNAs in different cancer types and neural stem/progenitor cells^9,18,26,33^. For instance, EZH2 has been shown to epigenetically regulate miR-218 and miR-200b in HCC, whereas BMI1 is known to be their downstream target^39–41^. In this study, we demonstrated that miR-200c was negatively regulated by EZH2, whereas both IHC and FISH analysis of the EZH2-depleted or control xenograft tumors also validated the EZH2-miR-200c-negative regulatory axis. Furthermore, we show that miR-200c overexpression inhibits the growth of huh7 cells in vivo and strongly antagonizes EZH2 dependent hepatocarcinogenesis. Mechanistically, luciferase reporter assay confirmed that miR-200c post-transcriptionally regulated BMI1 expression at protein level by binding to its 3′-UTR region. Consistent with this finding, recent studies show that miR-200c is frequently downregulated in a variety of tumors and often functions as a tumor suppressor microRNA^42–45^. In addition, we identify BMI1 as the functional target of miR-200c in HCC cells, since silencing miR-200c enhances the migration of HCC cells via upregulation of the BMI1 expression. Indeed, our previous study shows that knockdown of BMI1 dramatically inhibits the proliferation and migration in HCC cells^30^. Taken together, our result demonstrates for the first time that the EZH2-BMI1 signaling axis is linked by miR-200c in HCC cells.

As EZH2-mediated H3-K27 trimethylation acts as the functional upstream of PRC1^46^ and the expression level of BMI1 is highly correlated with patients prognosis in HCC^47^, we therefore investigate the correlation between their expression in HCC patients. Our clinical studies indicate that EZH2 and BMI1 are highly co-expressed in HCC patient samples. More importantly, the patients with high expression of EZH2 and BMI1 are associated with the worst OS in TCGA, suggesting that inhibition of EZH2 and BMI1 activity by small inhibitors can be a promising therapeutic strategy for HCC. The combination treatment of EZH2 and BMI1 inhibitors further repressed HCC cell viability as compared with the cells treated with either DMSO, EZH2, or BMI1 inhibitor alone.

In summary, our data demonstrate a novel mechanism whereby the alteration of EZH2 gene copy number values enhances its tumor expression, which then epigenetically silences miR-200c expression to up-regulate BMI1-dependent hepatocarcinogenesis, providing novel therapeutic targets for an improved HCC treatment.

## Materials and methods

### Clinical specimens

A cohort of 240 paraffin-embedded liver tumor samples as well as 25 paired frozen liver tumor and normal adjacent tissues were obtained from HCC patients undergoing surgery resection between 2011 and 2016 at Sun Yat-sen Memorial hospital (Guangzhou, China) with informed consent signed. This study has been approved by the ethics committee of Sun Yat-sen Memorial Hospital, Sun Yat-sen University.

### Cell lines and culturing conditions

Human HCC cell lines MHCC97H, HCCLM3, HCCLM6 were kindly provided by the Liver Cancer Institute of Fudan University (Shanghai, China). Human HCC cell lines huh7, hepG2, PLC/PRF5 were purchased from the America Type Culture Collection. Human HCC cell lines Li-7 and immortalized human liver cell HL-7702 were purchased from the Cell Bank of Typical Culture Preservation Committee of Chinese Academy of Science (Shanghai, China). The HCC cell lines were cultured at 37 °C in a cell incubator contain 5% CO_2_ in RPMI 1640 (BI, Israel) or Dulbecco’s modified Eagle’s medium (BI, Israel), supplemented with 10% fetal bovine serum (Gibco, America), 100 µg/ml streptomycin and 100 µg/ml penicillin (Hyclone). Cell lines used were authenticated and confirmed to be free of mycoplasma.

### Immunohistochemistry

IHC experiments were performed according to the standard procedures. The paraffin sections were immersed in ethylenediaminetetraacetic acid (pH = 9.0) and treated with microwave for EZH2 antigen retrieval, or immersed in Tris-HCl (pH = 9.2) and treated with high pressure for BMI1 antigen retrieval. Sections were incubated with primary antibodies: EZH2 1:800 (cell signaling technology, America), Bmi1 1:800 (cell signaling technology, America) overnight at 4 °C and then incubated with horseradish peroxidase-conjugated secondary antibody (DAKO, Denmark) 30 min in a 37°C air oven. Finally, the antibody binding was detected by DAB solutions and stained with hematoxylin for 30 s. The IHC scores were assessed via an image analysis workstation (Image Pro Plus 6.0, Media Cybernetics).

### Fluorescence in situ hybridization

FISH of miR-200c was conducted on the xenograft tumor and liver cancer paraffin sections. FISH was performed using miRNA fish kit A (GenePharma, China) and CY3-labeled miR-200c fluorescence probe (GenePharma, China). The paraffin sections were performed by deparaffinization, protease K treatment, denaturation, hybridization, washing, counterstaining with 4′,6-diamidino-2-phenylindole, and mounting according to the manufacturer suggested instruction.

### Cell transfection

Lipofectamine 3000 transfection kit (Invitrogen, America) was used for siRNAs, miRNA mimics, antagomir, and plasmid transfection. huh7 or hepG2 cells were seeded in a six-well plate at 3 × 10^5^ or 8 × 10^5^ per well. Afte 12–16 h later, HCC cells were transiently transfected with either siRNAs, miRNA mimics, antagomir, or plasmid according to the manufacturer’s instructions. In all, 24–48 h after transfection, the HCC cell were harvested for further experiments. All the sequences used for transient transfection were listed in Supplementary table S1.

### Establishment of EZH2 stable transfectant in HCC cell lines

The EZH2 knockdown and overexpression lentivirus as well as their corresponding negative control lentivirus were purchased from Hanbio Biotechnology and used according to the manufacturer’s instruction. In brief, huh7 and hepG2 cells were transfected with the lentivirus at a confluence of 30–50%. The HCC cell lines were infected with 1 mg/ml polybrene and lentivirus multiplicity of infection at 20 (hepG2) or 30 (huh7). More than 90% of cells were viable after transfection of 8 h and changed with fresh medium. After 3 days, 2 μg/ml puromycin was added and used to select successfully transfected cells. In all, 7–9 days later, the efficacy of infection was verified via western blot. The design of EZH2 knockdown and overexpression vectors used for this study were provide in the Supplementary table S2.

### Colony formation assay

HCC cells transfected with siRNA for 24 h were seeded into six-well plates at a concentration of 1000–2000 cells per well in complete growth medium. The medium was changed every 3 days. After 10 days of incubation, the cells were fixed with 4% paraformaldehyde for 20 min and stain with 1% crystal violet for 30 min. The colony number was counted using AID GmbH and every group was done in triplicate.

### Sphere formation assay

A total of 500–1000 cells/well were incubated in serum-free epithelial basal medium supplemented with B27 (5 × 50) (GIBCO, America), 20 ng/ml EGF (PeproTech, America) and 20 ng/ml FGF (PeproTech, America) in low-adhesion 96-plates. Fresh microsphere medium was regularly addicted to the 96-plate for supplement nutrient. After incubation for 7–14 days in cell incubator, the spheres were counted in the microscope. These experiments were done in triplicate.

### Transwell migration assay

In all, 6 × 10^4^ huh7 cells or 4 × 10^5^ hepG2 cells were seeded with serum-free medium in the upper compartment of transwell inserts (8 µm pore size, Corning, USA), whereas the lower bottom compartment was added with medium containing 20% fetal bovine serum. After 24 (huh7) or 72 h (hepG2) of incubation in cell incubator, the non-migration cells on the upper chamber were removed. Migrated cells adhering to the lower chamber were fixed with 4% paraformaldehyde for 20 min and stained with 0.1 crystal violet for 30 min. The experiments were repeated in triplicate and in each experiment three random fields were selected for cell quantification.

### CCK8 proliferation assay and cell counting experiment

CCK8 proliferation assay kits (YEASEN, China) were used to estimate the cell proliferation in vitro. Human HCC cells were seeded into 96-well plate at a density of 1000–2000 cell/well. After the cultured cells grow against the wall of flack, the medium was changed with complete medium containing 10% CCK8 solution at different time point (day 0, 2, 4, 6, or day 1, 3, 5, 7). After incubation for 1–2 h in incubator, the absorbance at 450 nm wavelength was measured in TS Microplate Reader. For cell proliferation assay, the transfected huh7 and hepG2 were seeded into 12-well plate at a density of 5000 and 10,000 cell/well, respectively. The cell numbers were counted using countstar on day 1, 3, 5, and 7. These experiments were done in triplicate.

### Luciferase reporter assay

Huh7 and hepG2 cells were plated into a 12-well plate at a density of 1.5 × 10^5^ and 4 × 10^5^ per well, respectively, and grown overnight. The next day, HCC cell lines were co-transfected with psicheck2 dual luciferase report vector (300 ng per well) and miRNA mimics (40 nM) or antagomirs(100 nM) or pGL3 luciferase reporter (500 ng per well), pRL-TK reference plasmid (200 ng per well) and siRNA(60 nM) using lipofectamine 3000 (Invitrogen, America). In all, 6–8 h later, the medium were changed with fresh complete medium. After 24–48 h of transfection, the luciferase activity was measured by using Dual-Glo^®^ Luciferase Assay System (Promega, America) according to the manufacturer’s instructions. These experiments were done in triplicate.

### Western blotting

Huh7 and hepG2 cells were harvested and lysed with RIPA buffer (cwbiotech, China) supplemented with 1× protease inhibitor (cwbiotech, China). Protein concentration was measured by bicinchoninic acid (Invitrogen, America). First, the protein was separated by 12% sodium dodecyl sulfate polyacrylamide gel electrophoresis Gel (Solarbio, China) and transferred to PVDF membrane (Millipore, America). Then, the membrane was blocked with 5% skimmed milk in 1× tris-buffered saline and Polysorbate 20 for 1 h at room temperature and incubated with specific primary antibody, including anti-EZH2 (1:2000; Cell signaling Technology), anti-BMI1 (1:2000; Cell signaling Technology), anti-GAPDH (1:2000; Cell signaling Technology), anti-H3K27me3 (1:1000; Cell signaling Technology), anti-H3 (1:1000; Cell signaling Technology), at 4 °C overnight. The following day, the membrane was incubated with horseradish peroxidase labeled as goat anti-rabbit immunoglobulin G (1:10,000; Cell signaling Technology) at room temperature for 1 h. Finally, the membrane was exposure with ECL (Millipore, America). All the western blots were performed in three independent replicates. Relative densitometry was calculated by Image J analysis software.

### RNA isolation and RT-qPCR

Total RNA were extracted from HCC cells or frozen liver tissues using RNAiso Plus (Takara, Japan). The reverse transcription (RT) and qPCR were performed with Mir-X^TM^ miRNA First-Strand Synthesis, PrimeScript RT Master Mix and SYBR Premix Ex Taq II (Takara) according to the manufacturer’s instruction. Each experiment was performed in triplicate. RT-qPCR of miR-200c detection was done as previously described^40,48,49^. U6 and GAPDH were used as housekeeping genes normalization for miR-200c and other transcripts, respectively, and the relative expression level was calculated with 2^^−ΔΔCt^ methods. The primers were list in the Supplementary table S3.

### Xenograft mouse model

The animal experiment was performed according to the guidelines for laboratory animal care of South China University of Technology and all the 10–14 mice were randomly divided into two groups by lottery. And then, 5 × 10^6^ huh7 cells stably transfected with scramble control vector (LV-shCtrl/LV-miR-C) or shRNA targeting EZH2 (LV-shEZH2) or miR-200c expressing lentivirus (LV-miR-200c) were re-suspended in 100 μL PBS–matrigel mixture (PBS: Matrigel at 1:1) and then injected subcutaneously into male athymic nude mice (4–5 weeks old). The animals that died of other diseases before the end of the experiment would be excluded from the final analysis. In all, 3–4 weeks later, the mice were killed and tumors excised, imaged, measured with Vernier caliper and weighted. The tumor volumes were calculated as 1/2 × width × width × length.

### Quantification and statistical analysis

All statistical analysis was performed using Prism 8 software package and IBM SPSS 20. Mann–Whitney test was used to determine the correlations between gene expression and clinicopathological parameters. Log-rank (Mantel–Cox) tests were used for OS and DFS. Pearson correlation test was used to analyze the correlation among EZH2, miR-200c and BMI1 in HCC tissues. The data obtained from in vitro and vivo experiments were compared with one-way analysis of variance (ANOVA), two-way ANOVA, Kruskal–Wallis test or Student’s *t* test according to the corresponding data type. All the experimental data were present in the mean±standard error. *p* < 0.05 was considered as significant difference.

### Animal ethical regulations

All animal procedures were approved by the institutional Ethics Committee on Ethics of South China University of Technology.

## Supplementary information

Supplemental figure 1

Supplemental figure 2

Supplemental figure 3

Supplemental figure 4

Supplemental figure 5

Supplemental figure and table legend

Supplemental table 1

Supplemental table 2

Supplemental table 3
