# Supplementary figures and images for: EZH2 facilitates BMI1-dependent hepatocarcinogenesis through epigenetically silencing microRNA-200c
Source: Oncogenesis. 2020 Nov 9;9(11):101. doi: 10.1038/s41389-020-00284-w (PMC7652937; doi:10.1038/s41389-020-00284-w)

Figure S1

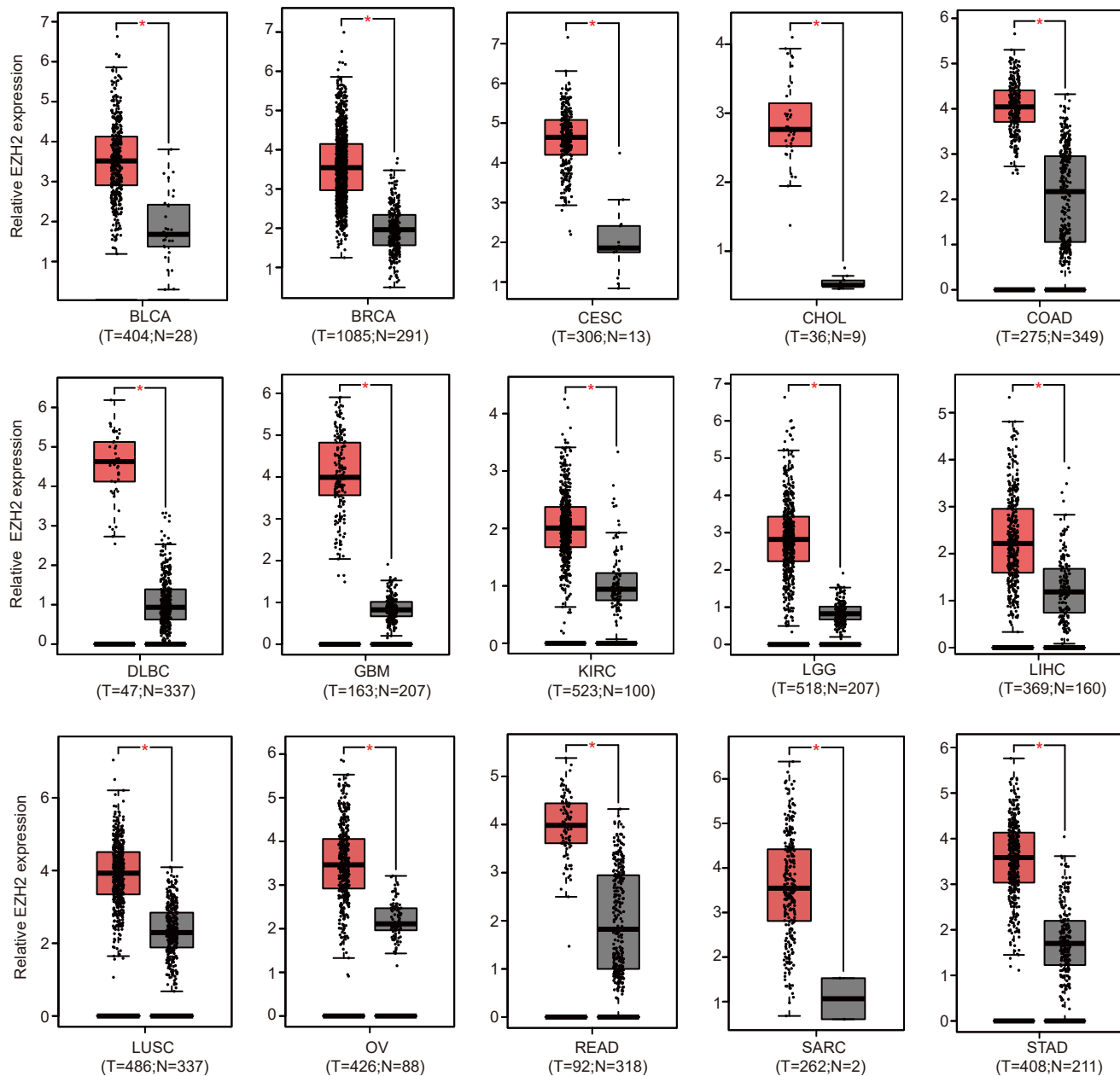

Supplement: Supplementary file 1 — Supplemental figure 1 [file 41389_2020_284_MOESM1_ESM.pdf]

Figure S2

A

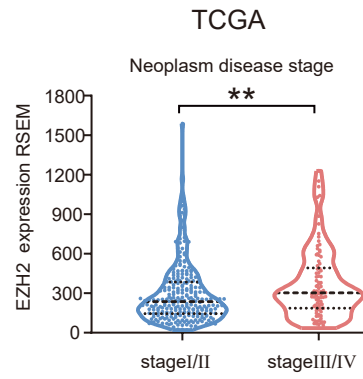

B

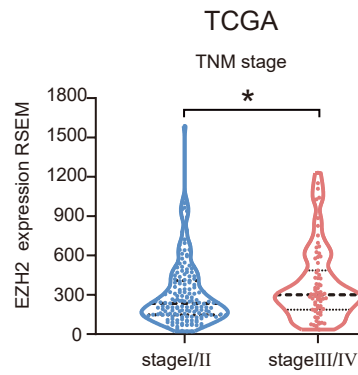

C

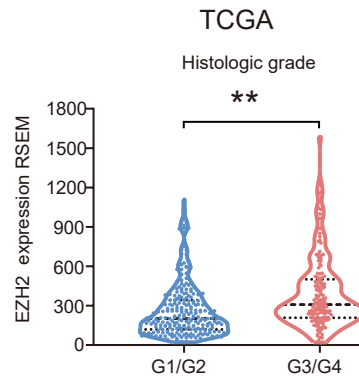

Supplement: Supplementary file 2 — Supplemental figure 2 [file 41389_2020_284_MOESM2_ESM.pdf]

Figure S3

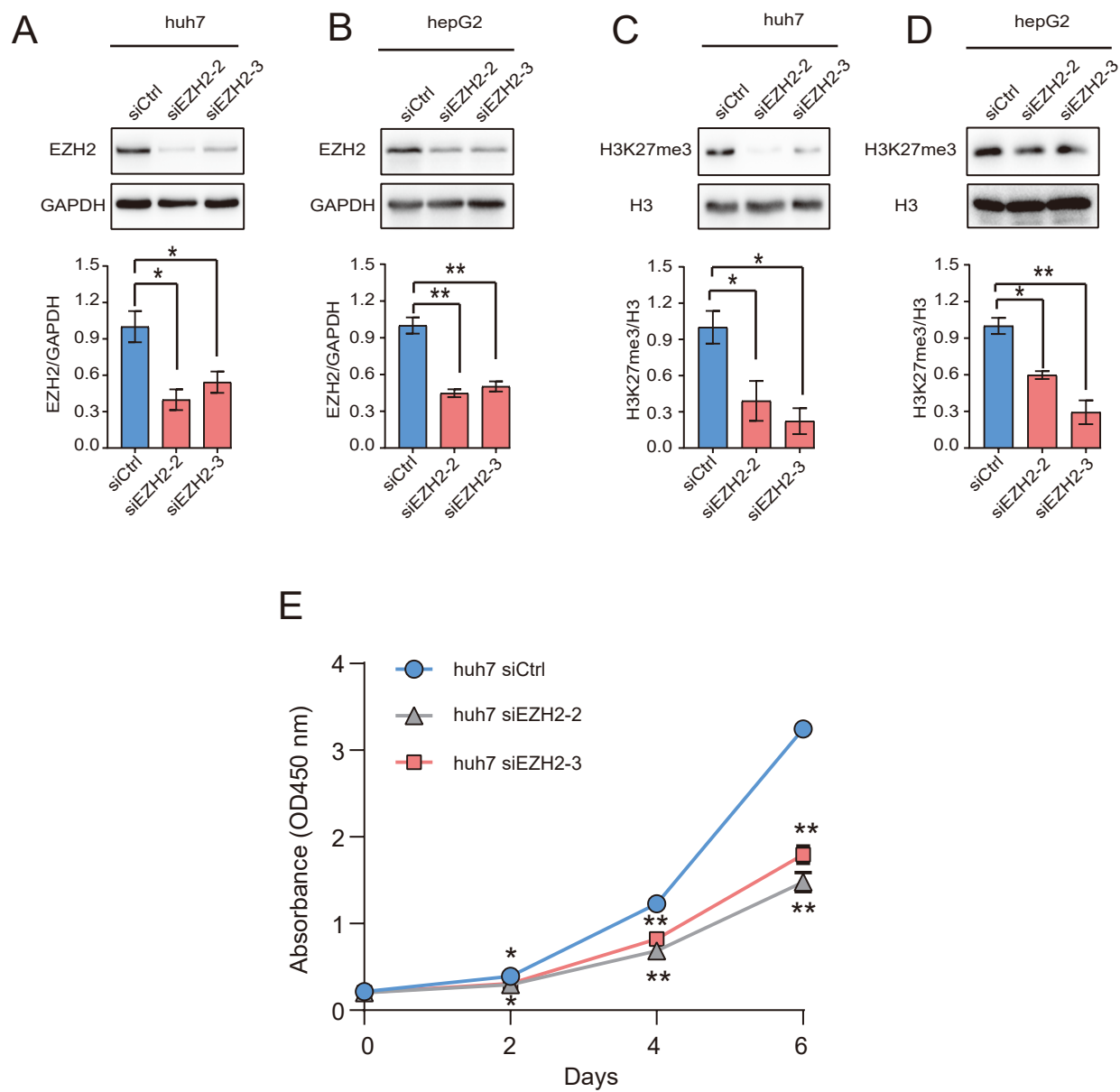

Supplement: Supplementary file 3 — Supplemental figure 3 [file 41389_2020_284_MOESM3_ESM.pdf]

Figure S4

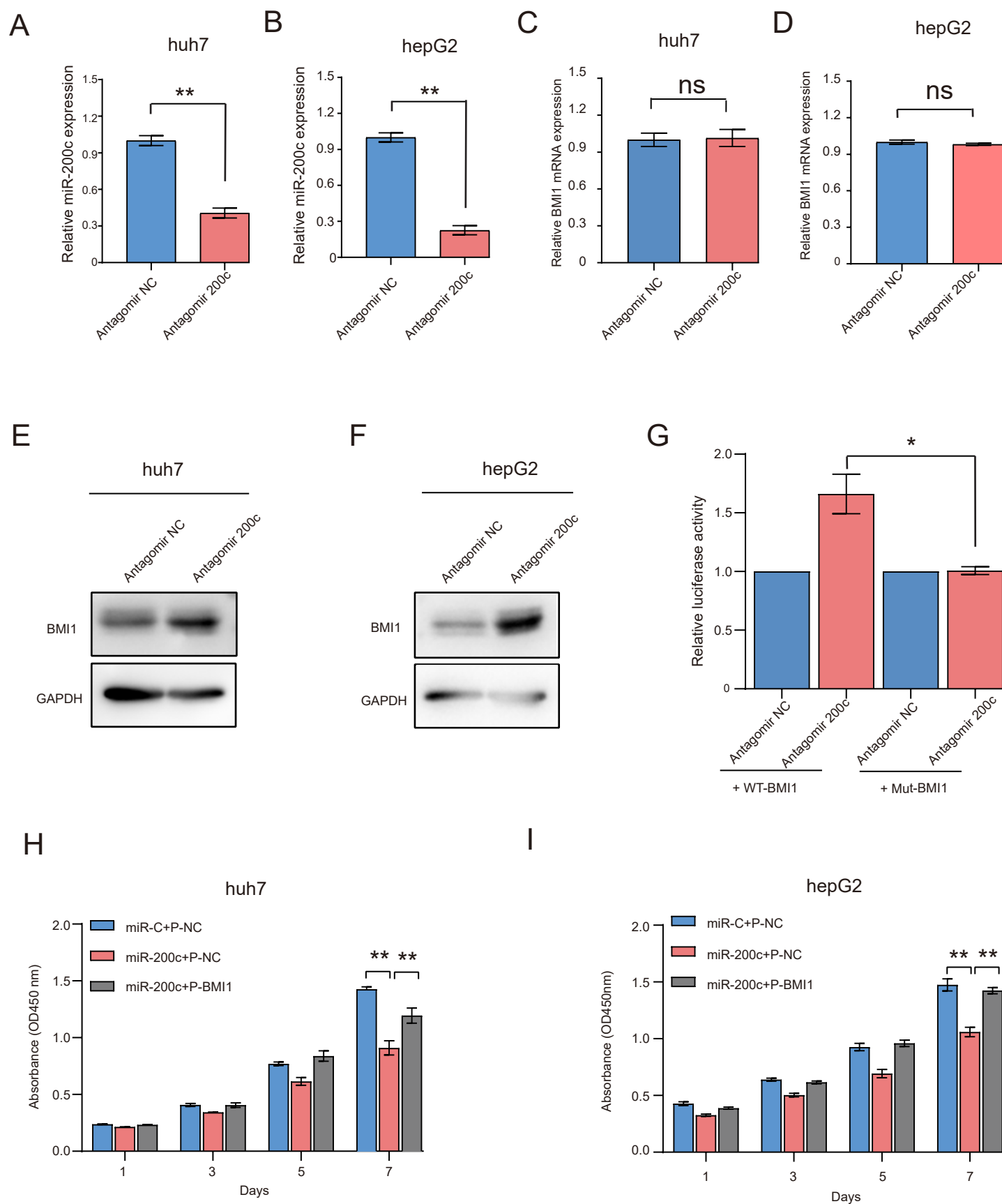

Supplement: Supplementary file 4 — Supplemental figure 4 [file 41389_2020_284_MOESM4_ESM.pdf]

Figure S5

EZH2 inhibitor

A

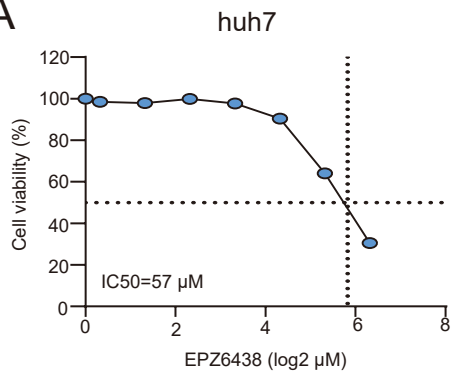

B

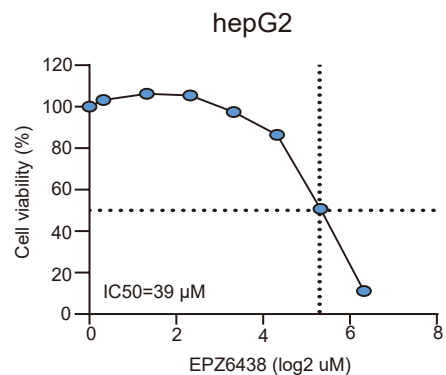

BMI1 inhibitor

C

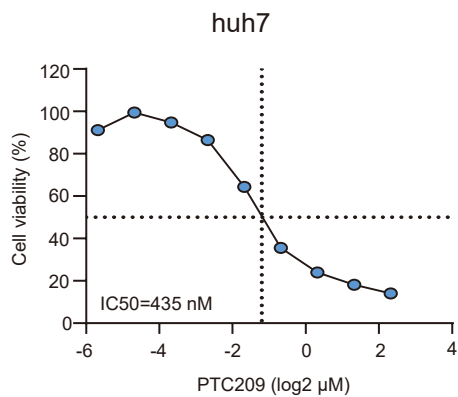

D

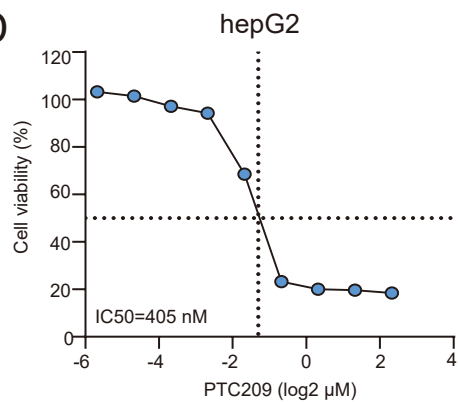

Supplement: Supplementary file 5 — Supplemental figure 5 [file 41389_2020_284_MOESM5_ESM.pdf]
