# Supplementary material for: EZH2 facilitates BMI1-dependent hepatocarcinogenesis through epigenetically silencing microRNA-200c
Source: Oncogenesis. 2020 Nov 9;9(11):101. doi: 10.1038/s41389-020-00284-w (PMC7652937; doi:10.1038/s41389-020-00284-w)
Supplement: Supplementary file 6 — Supplemental figure and table legend [file 41389_2020_284_MOESM6_ESM.doc]

**Supplementary figure and table legend**

**Figure S1. EZH2 expression is frequently up-regulated in multiple cancers.** GEPIA database analysis of the relative mRNA expression of EZH2 in matched cancer tissues and adjacent normal tissues from different cancer types, including Bladder Urothelial Carcinoma (BLCA), Breast invasive carcinoma (BRCA), Cervical squamous cell carcinoma and endocervical adenocarcinoma (CESC), cholangiocarcinoma (CHOL), colon adenocarcinoma (COAD), diffuse large B-cell lymphoma (DLBC), glioblastoma multiforme (GBM), kidney renal clear cell carcinoma (KIRC), brain lower grade glioma (LGG), liver hepatocellular carcinoma (LIHC), lung squamous cell carcinoma (LUSC), ovarian serous cystadenocarcinoma (OV), rectum adenocarcinoma (READ), sarcoma (SARC), stomach adenocarcinoma (STAD). One-way ANOVA was used for differential analysis. Red box colour represents tumors and grey box colour for adjacent normal tissues. T represents tumors and N for adjacent normal tissues.

**Figure S2. Expression of EZH2 is associated with neoplasm disease stage, cancer progression and histologic grade in HCC patients.** Analysis of EZH2 expression in human hepatocellular carcinomas from TCGA dataset (N = 366 patients). **A-C**, High expression of EZH2 stratified the HCC patients into those with elevated neoplasm disease stage (**A**), advanced cancer progression (**B**), increased histologic grade (**C**). Each sample on the violin plots represents individual patient data. **p* < 0.05, ***p* < 0.01. (**A-C**) Mann-Whitney test.

**Figure S3. Silencing EZH2 prohibits the proliferation of HCC cells.** Huh7 and hepG2 were transiently transfected with siCtrl, siEZH2-2 or siEZH2-3 and used for subsequent assays. **A-D**, Western blot analysis of the EZH2 and H3K27me3 levels in huh7 and hepG2 cells transiently transfected with either scramble (siCtrl), siEZH2-2 or siEZH2-3 for 48 h. GAPDH and total Histone 3 were used as a loading control respectively. All the western blots were performed at three independent replicates. Relative densitometry was calculated by Image J software. **E,** CCK8 proliferation assay of the huh7 cells transiently transfected with either siCtrl, siEZH2-2 or siEZH2-3. The data were obtained from three independent experiments and presented with mean ± S.E.M. * *p*<0.05, ** *p*<0.01. (**A-D**) One-way ANOVA. (**E**)Two-way ANOVA.

**Figure S4. Depletion of microRNA-200c increases the expression of BMI1 at protein level but not at mRNA level in HCC cell lines.** Huh7 and hepG2 were transiently transfected with antagomir NC or antagomir 200c and used for subsequent assays. **A, B,** RT-qPCR analysis of the relative expression of miR-200c in huh7 (**A**) and hepG2 cells (**B**). **C, D,** RT-qPCR analysis of the relative mRNA expression of BMI1 in huh7 (**C**) and hepG2 cells (**D**). **E, F,** Western blot analysis of the relative protein expression of BMI1 in huh7 (**E**) and hepG2 cells (**F**). **G,** Luciferase reporter assay of hepG2 after co-transfected with the psicheck2 dual luciferase reporter vector containing the insert of either the predicted wild-type-3’-UTR (WT-BMI1) or mutated-3’-UTR region (Mut-BMI1) of BMI1 together with antagomir NC or antagomir 200c. **H-I,** CCK8 proliferation assay of the Huh7 (**H**) and HepG2 cells (**I**) stably transfected with either vector conrol or miR-200c lentivirus, together with transient transfection of P-NC or P-BMI1 vector. The data were obtained from three independent experiments and presented with mean ± S.E.M. ** *p*<0.01. ns, no significant difference. (**A-G**) Student’s *t* test. (**H-I**) Two-way ANOVA.

**Figure S5. Treatment with an EZH2 inhibitor EPZ6438 or BMI1 inhibitor PTC209 prohibits the viability of huh7 and hepG2 cells. A-D,** The IC_50_ doses of EZH2 and BMI1 small molecule inhibitors were determined in huh7 and hepG2.

**Supplementary table S1. The sequences of RNAi, miRNA mimics and antagomir used in this study.**

**Supplementary table S2. The sequences of shRNA used in this study.**

**Supplementary table S3. The primer sequences used in this study.**
