# Supplementary material for: EZH2 facilitates BMI1-dependent hepatocarcinogenesis through epigenetically silencing microRNA-200c
Source: Oncogenesis. 2020 Nov 9;9(11):101. doi: 10.1038/s41389-020-00284-w (PMC7652937; doi:10.1038/s41389-020-00284-w)
Supplement: Supplementary file 7 — Supplemental table 1 [file 41389_2020_284_MOESM7_ESM.doc]

Supplementary table S1 The sequences of RNAi, miRNA mimics and antagomir used in this study.

| name | sequence |  |
| --- | --- | --- |
| NC | UUCUCCGAACGUGUCACGUTT | ACGUGACACGUUCGGAGAATT |
| EZH2-homo-614 | GGAUGGUACUUUCAUUGAATT | UUCAAUGAAAGUACCAUCCTT |
| EZH2-homo-2167 | GAGGGAAAGUGUAUGAUAATT | UUAUCAUACACUUUCCCUCTT |
| antagomirNC | CAGUACUUUUGUGUAGUACAA |  |
| antagomir200c | UCCAUCAUUACCCGGCAGUAUUA |  |
| miR-200c mimics | UAAUACUGCCGGGUAAUGAUGGA | CAUCAUUACCCGGCAGUAUUAUU |
