# Supplementary material for: EZH2 facilitates BMI1-dependent hepatocarcinogenesis through epigenetically silencing microRNA-200c
Source: Oncogenesis. 2020 Nov 9;9(11):101. doi: 10.1038/s41389-020-00284-w (PMC7652937; doi:10.1038/s41389-020-00284-w)
Supplement: Supplementary file 8 — Supplemental table 2 [file 41389_2020_284_MOESM8_ESM.doc]

Supplementary table S2 The sequences of shRNA used in this study.

| Name | 5'-3' |
| --- | --- |
| shCtrl | GATCCGTTCTCCGAACGTGTCACGTAATTCAAGAGATTACGTGACACGTTCGGAGAATTTTTTC |
| shEZH2-1 | GATCCGCCCAACATAGATGGACCAAATTTCAAGAGAATTTGGTCCATCTATGTTGGGTTTTTTG |
| shEZH2-2 | GATCCGACTCTGAATGCAGTTGCTTCAGTATTCAAGAGATACTGAAGCAACTGCATTCAGAGTCTTTTTTG |
| shEZH2-3 | GATCCGGAATCAAAGGATACAGACAGTGATTTCAAGAGAATCACTGTCTGTATCCTTTGATTCCTTTTTTG |
