# Supplementary material for: EZH2 facilitates BMI1-dependent hepatocarcinogenesis through epigenetically silencing microRNA-200c
Source: Oncogenesis. 2020 Nov 9;9(11):101. doi: 10.1038/s41389-020-00284-w (PMC7652937; doi:10.1038/s41389-020-00284-w)
Supplement: Supplementary file 9 — Supplemental table 3 [file 41389_2020_284_MOESM9_ESM.doc]

Supplementary table S3 The primer sequences used in this study.

| Primer | Forward (5' - 3') | Reverse (5' - 3') |
| --- | --- | --- |
| miR-200c | TACTGCCGGGTAATGATGGA | Universal primer |
| U6 | GGAACGATACAGAGAAGATTAGC | TGGAACGCTTCACGAATTTGCG |
| EZH2 | GACATACTTCAGGGCATCAGC | TCATTCGGTAAATCCAAACTG |
| BMI1 | CCCTCCACCTCTTCTTGTTTGC | ATGACCCATTTACTGATGATTTTCG |
| GAPDH | CGCTGAGTACGTCGTGGAGTC | GCTGATGATCTTGAGGCTGTTGTC |
